# Evaluation of artificial intelligence techniques in disease diagnosis and prediction

**DOI:** 10.1007/s44163-023-00049-5

**Published:** 2023-01-30

**Authors:** Nafiseh Ghaffar Nia, Erkan Kaplanoglu, Ahad Nasab

**Affiliations:** College of Engineering and Computer Science, The University of Tennessee at Chattanooga, Chattanooga, TN 37403 USA

**Keywords:** Artificial intelligence, Deep learning, Machine learning, Diseases diagnosis, Medical image processing

## Abstract

A broad range of medical diagnoses is based on analyzing disease images obtained through high-tech digital devices. The application of artificial intelligence (AI) in the assessment of medical images has led to accurate evaluations being performed automatically, which in turn has reduced the workload of physicians, decreased errors and times in diagnosis, and improved performance in the prediction and detection of various diseases. AI techniques based on medical image processing are an essential area of research that uses advanced computer algorithms for prediction, diagnosis, and treatment planning, leading to a remarkable impact on decision-making procedures. Machine Learning (ML) and Deep Learning (DL) as advanced AI techniques are two main subfields applied in the healthcare system to diagnose diseases, discover medication, and identify patient risk factors. The advancement of electronic medical records and big data technologies in recent years has accompanied the success of ML and DL algorithms. ML includes neural networks and fuzzy logic algorithms with various applications in automating forecasting and diagnosis processes. DL algorithm is an ML technique that does not rely on expert feature extraction, unlike classical neural network algorithms. DL algorithms with high-performance calculations give promising results in medical image analysis, such as fusion, segmentation, recording, and classification. Support Vector Machine (SVM) as an ML method and Convolutional Neural Network (CNN) as a DL method is usually the most widely used techniques for analyzing and diagnosing diseases. This review study aims to cover recent AI techniques in diagnosing and predicting numerous diseases such as cancers, heart, lung, skin, genetic, and neural disorders, which perform more precisely compared to specialists without human error. Also, AI's existing challenges and limitations in the medical area are discussed and highlighted.

## Introduction

**Fig.1:**
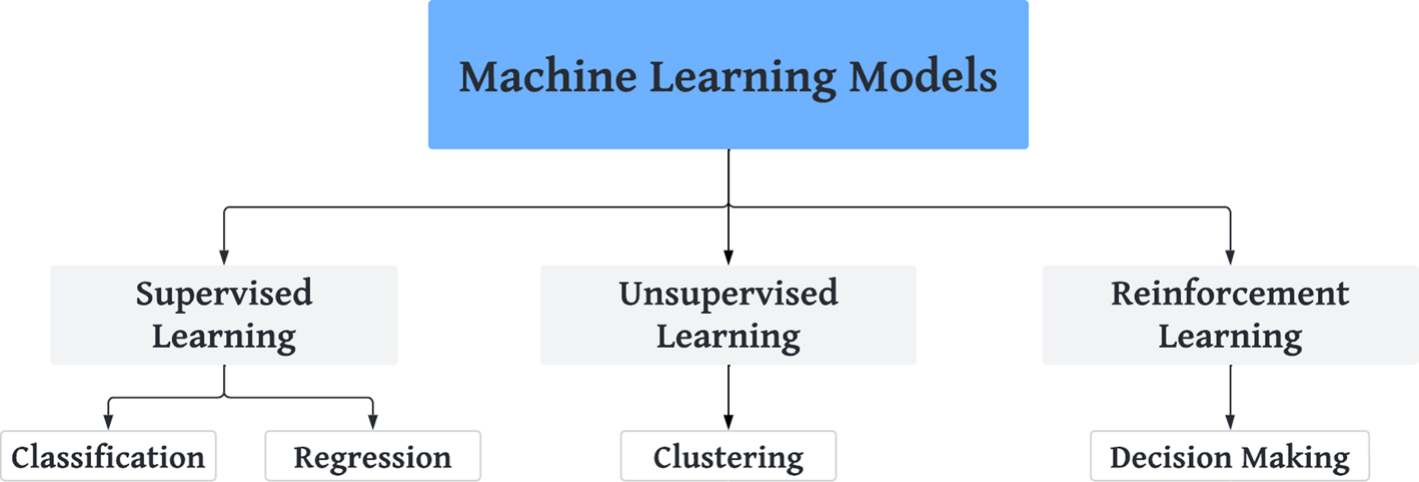
Machine Learning models and main algorithms

Advances in emerging computer-based technologies are overgrowing. Digital healthcare offers numerous opportunities to reduce human error, improve clinical outcomes, and track data over time. Artificial Intelligence (AI) methods, including Machine Learning (ML) and Deep Learning (DL) algorithms, are widely used in the prediction and diagnosis of several diseases, especially those whose diagnosis is based on imaging or signaling analysis [1, 2]. AI can also help to identify demographics or environmental areas where disease or high-risk behaviors are prevalent [3, 4]. ML techniques have achieved significant success in medical image analysis due to the advanced algorithms that enable the automated extraction of improved features [5, 6]. ML is based on learning methods and can be divided into three categories: supervised (classification, regression, and composition), unsupervised (association, clustering, and dimensionality), and reinforced learning [7] (Fig. 1).

**Fig. 2:**
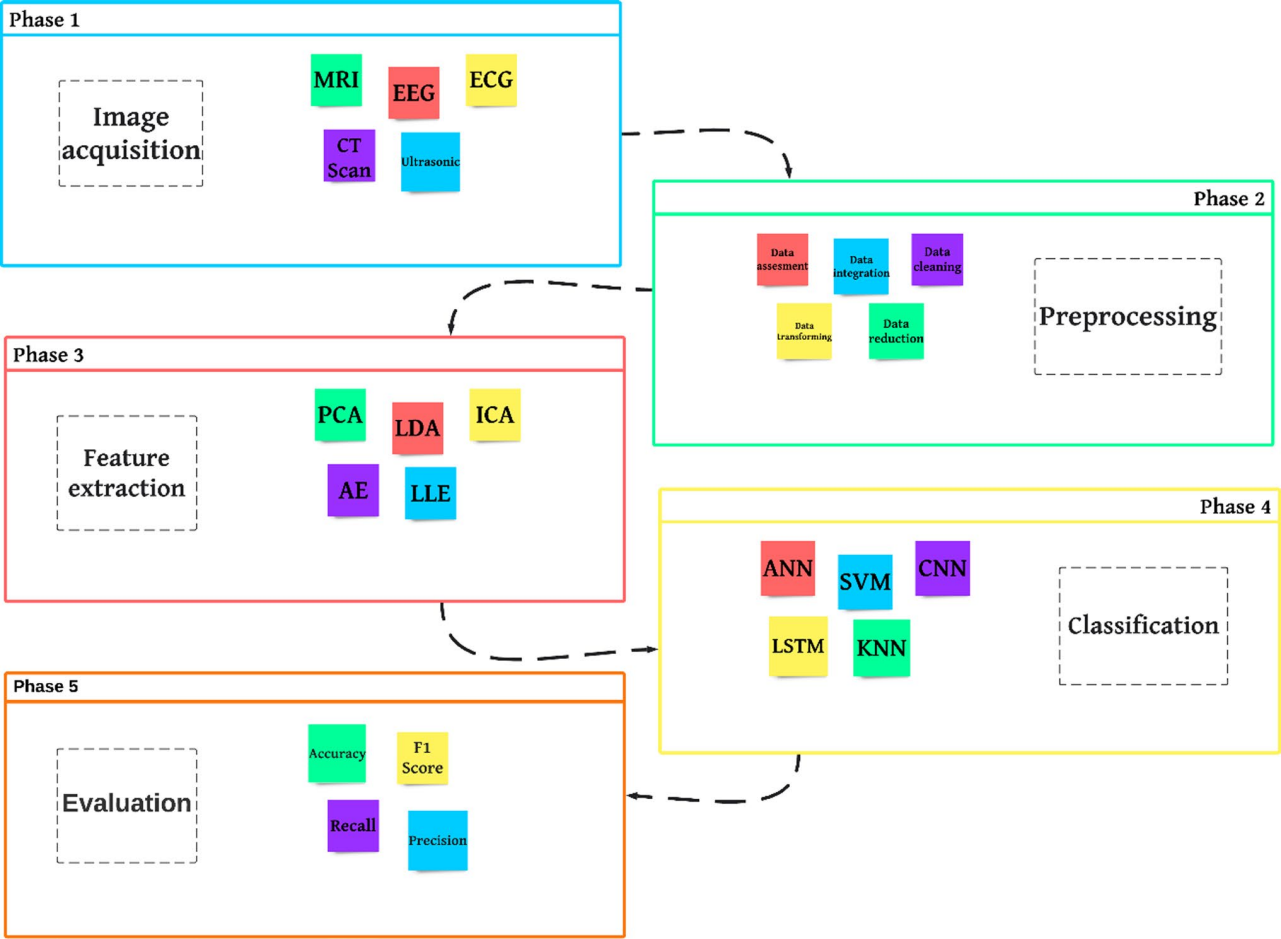
The classification process phases in medical image analysis

Several computations and operations on input data are performed through ML algorithms. Data preprocessing is the first and essential step to reducing false predictions or incorrect results, speeding up the data processing, and eventually improving overall data quality. After data preprocessing, crucial features are extracted and implemented according to the selected ML or DL model for image classification. Feature selection reduces dimension and boosts algorithm performance [8]. Model training and parameter adjustment are also performed based on the chosen algorithm through data processing to make accurate decisions and obtain reasonable classifications or predictions in the last phase, Fig. 2.

ML enables computers to perform the tasks of medical professionals. ML has a widely used subfield in medical image recognition called deep learning (DL). DL is a method for designing the ML algorithm in which simple concepts are built on top of each other to form a deep structure with numerous processing layers. In other words, DL is the development of ML for analyzing massive data [9–11]. It replaces the classical manual method of designing and extracting patterns used for classification with an automated strategy that allows a computer to decide which features are essential by training on a dataset. While DL is not a new concept, processing massive data and increased computing power have made DL successful and popular in recent years.

DL has outperformed previous advanced algorithms in several visual recognition tasks, and its performance has improved significantly. ImageNet Large Scale Visual Recognition Challenge [12] is annually the largest object recognition competition. Researchers classified the 1.3 million high-resolution images using the DL-based CNN model [13]. They dramatically improved their model performance by achieving error rates of 39.7% and 18.9% and winning the challenge. DL algorithms have become more popular since then. A DL algorithm is a deep artificial neural network (ANN) inspired by human brain cells that consist of several simple processing units that combine to form a more complex architecture. These units are grouped in layers in every algorithm and referred to as nerve cells, where the input signals are combined and transferred to other cells if their value is higher than the threshold value. In the synthetic type, they are replaced by a sum and an activation function that combine to create more complex relationships, similar to the human brain, through the network.

**Fig. 3:**
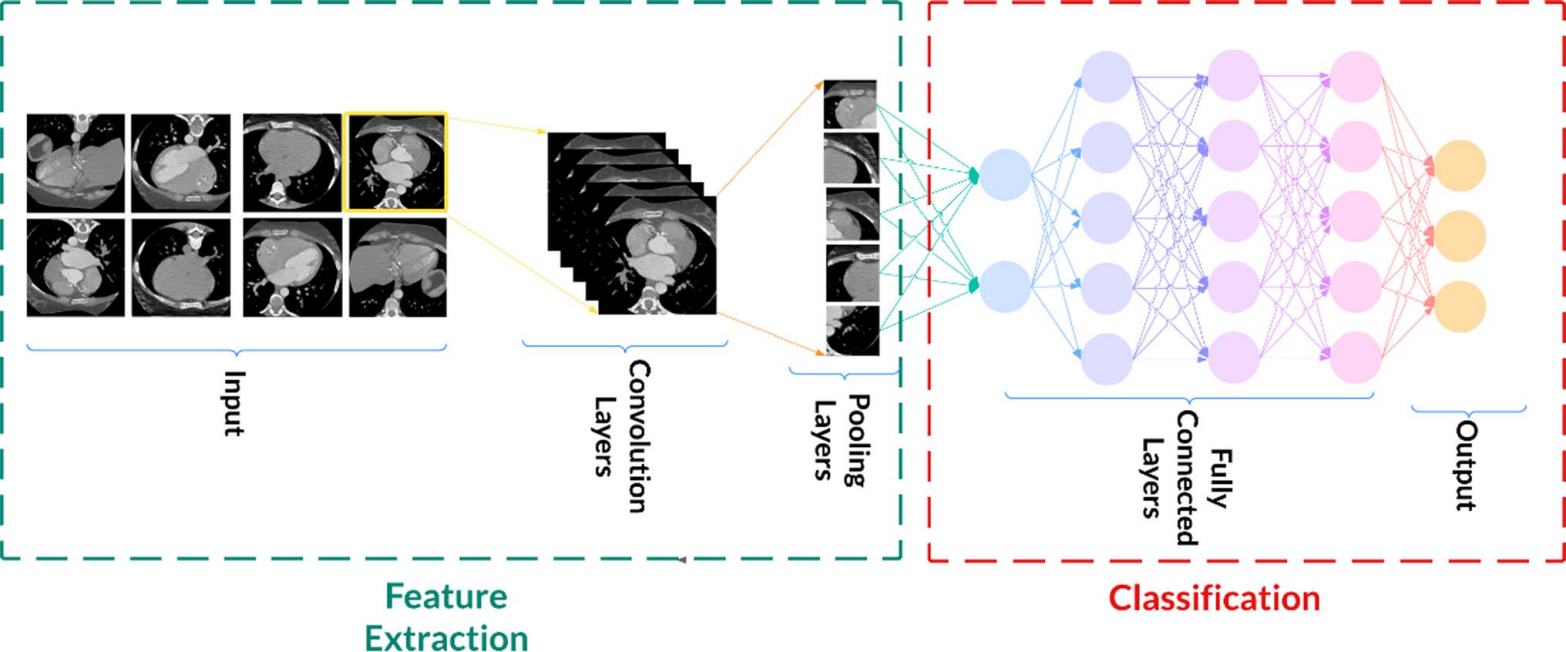
The DL model (CNN) structure for classification of brain disease using brain CT scan images as input data

A convolution neural network (CNN), as a successful approach for image analysis and classification, is a supervised DL model. CNN consists of fully connected layers with standard weights that lead to fewer parameters for training features through the backpropagation process. They are designed to extract spatial information from input images. CNN aims to learn hierarchical features adaptively, classify image data, and extract their features automatically, as demonstrated in Fig. 3. The essential advantage of this algorithm is learning very abstract features with few parameters and simple preprocessing.

The neural network's initialization and the samples' order during the training phase are usually random. However, when the training is over, there is nothing unexpected with the neural network. It involves well-defined calculations, but its complex and profound structure often make it incomprehensible to humans. Therefore, the training method is usually not mentioned to explain the behavior of networks. Typically, a trained network can be understood by the characteristics of the data set used for training.

A large amount of data is processed in the healthcare system, making it a suitable platform for developing successful algorithms that can be exploited using DL or ML approaches. While many different data types are used for medical evaluations, images are a broad data type that makes medical analytics potentially very tangible. Medical images are created in other forms using various equipment, such as ultrasound, X-rays, computed tomography, magnetic resonance imaging, microscopy, and scintigraphy. All these techniques can create diverse images, but all images have the potential for auto-analysis through AI algorithms to investigate and predict various diseases. To understand how each AI-based model helps diagnose and predict disease, examining the application of multiple algorithms is essential [14, 15].

Recently, ML and DL techniques have established an advanced approach to emerging techniques in computer-based diagnosis, which have been widely conducted in various medical fields to diagnose or predict multiple diseases [16–19]. These methods have led to more accurate diagnoses and increased efficiency [16, 17]. This paper reviews the ML and DL models for diagnosing various diseases.

## Related works

AI has recently undergone significant advances that have achieved much attention from numerous companies and academic fields. The most successful technique is driven by advances in ANNs, called Deep Learning (DL), a set of processes and algorithms that automatically enable computers to detect complex patterns in large datasets. Feeding these advances is increased access to data (“big data”), user-friendly software frameworks, and an explosion of existing computing power that allows deep neural networks to be widely used. DL became prominent in image processing when neural networks performed better than other methods in several high-resolution image analysis criteria.

In the ImageNet Large-Scale Visual Recognition Challenge (ILSVRC) [12], a CNN model reduced the second-highest error rate in image classification work by 50% in 2012. Before that, computers were thought to be very difficult to detect objects in natural images. So far, CNN has even surpassed human performance in ILSVRC to the point where the task of classifying ILSVRC is essentially solved. DL techniques have become the objective standard solution for various computer vision problems. Numerous studies have suggested the use of DL techniques in the diagnosis of acute human diseases.

Researchers have used multiple scenarios based on ML and DL models to predict conditions such as liver disease, heart disease, Alzheimer's disease, and various types of cancers for which early detection is vital for treating [20–22]. Some researchers have used DL techniques to diagnose and differentiate bacterial pneumonia using pediatric chest radiographs [23, 24]. Significant efforts have also been made to identify the different features of chest CT imaging characteristics of various diseases [25, 26]. New hybrid models based on Case-Based Reasoning were proposed to diagnose various skin diseases in different studies [27, 28]. The model’s output as an application could diagnose multiple skin diseases and suggest proper treatment. Proposing personalized real-time monitoring systems based on ANN techniques to receive vital information about the body is widely used in healthcare. This device can help patients manage their health, especially in critical conditions [29]. Researchers in [30] applied ANN models for predicting diabetes disease and achieved 91% accuracy.

ML classification methods are implemented to analyze data automatically for the diagnosis or prediction of various diseases. Researchers developed a personalized real-time web-based healthcare monitoring system to receive vital information from the body, such as heart rate or blood pressure. This device can help patients manage their health, especially in critical conditions [31].

AI approaches combined with the Internet of Things (IoT) method in the healthcare system can upgrade treatment procedures and healthcare technology. A reliable IoT-based system using ML algorithms for healthcare was proposed to monitor human activities and the surrounding environment through the body sensor network, BSN-Care [32]. Another study suggested a hybrid IoT model using a healthcare monitoring system and the Random Forest technique to predict type 2 diabetes (T2D) [33]. Researchers also investigated the risk of T2D among people based on their personal lifestyle information and achieved high accuracy using the random forest classifier, which outperformed other algorithms [34]. A mobile-based platform was developed for real-time tuberculosis disease (TD) antigen-specific antibody detection using the random forest classifier and gained 98.4% accuracy [35]. A research study proposed an AI-based framework for classifying multiple gastrointestinal (GI) diseases using RNN and LSTM networks and achieved 97.057% accuracy [14].

Hypertension healthcare control and awareness are the two most critical points to reducing stroke and cardiovascular disease. Researchers assessed digital healthcare technologies and AI in this regard and suggested a privacy protection system to collect and store individuals' data [36]. Furthermore, many researchers have done several studies on disease prediction to recognize and predict them in their early stages. A novel hybrid ML model was proposed based on the IoT for detection in the initial phase of diseases with an accuracy of 100% and a precision of 99.50% [37]. In another work, researchers have proposed an approach to predict cardiovascular disease according to various features. They used a hybrid random forest classifier and gained 88.7% accuracy [38].

A research study on detecting positive urine culture proposed an ML algorithm, XGBoost, to accurately diagnose results [39]. This model outperformed other developed models, and its accuracy ranged from 0.826 to 0.904. Another study used the CNN model for feature extraction in malaria-infected blood cell images [40]. Another work also predicted malaria infection using an ML model [41]. Researchers used different ML algorithms such as Random Forest, Support Vector Machine, Logistic Regression, K-Nearest Neighbor, and Naïve Bayes to identify acute exacerbations in chronic obstructive pulmonary disease. They found the SVM model achieved the best performance [42]. In other research work, scientists used three ML algorithms, including ANN, distributed random forest, and gradient boosting, to predict opioid abuse in adolescents based on the 2015–2017 National Survey on Drug Use and Health data. The prediction performance for the area under the receiver operating characteristic curve (AUROC) values ranges from 0.809 to 0.815 [43]. Likewise, other researchers used multiple ML algorithms, such as CNN, RF, SVM, DT, and AdaBoost classifiers, to propose a model for detecting COVID-19 from an X-ray image dataset and achieved a result of 98.91% accuracy [44]. ML and DL techniques can be used to detect stress levels in individuals. One approach is to use physiological signals, such as heart rate or respiration, to detect stress. For example, in an extensive study, authors examined different ML models for stress levels based on heart rate variability [45]. In this work, ML Random Forest outperformed other methods. Other researchers used various ML models to predict diabetes. In their work, Logistic Regression and Support Vector Machines performed well [46]. In a comprehensive study, researchers used different ML models such as KNN, SVM, ANN, Decision Tree, Logistic Regression, Naïve Bayes, Random Forest, and XGBoost to predict the risk of chronic type 2 diabetes [47]. In this study, the Random Forest model overpassed the other models with 0.91AUC. Recently, an extended DL model called 3DCellSeg provided powerful performance for analyzing and separating image-based diseases compared to basic models. This DL method has a lightweight deep CNN and requires only one hyperparameter [48].

## Artificial intelligence techniques in disease diagnosis and prediction

**Fig. 4:**
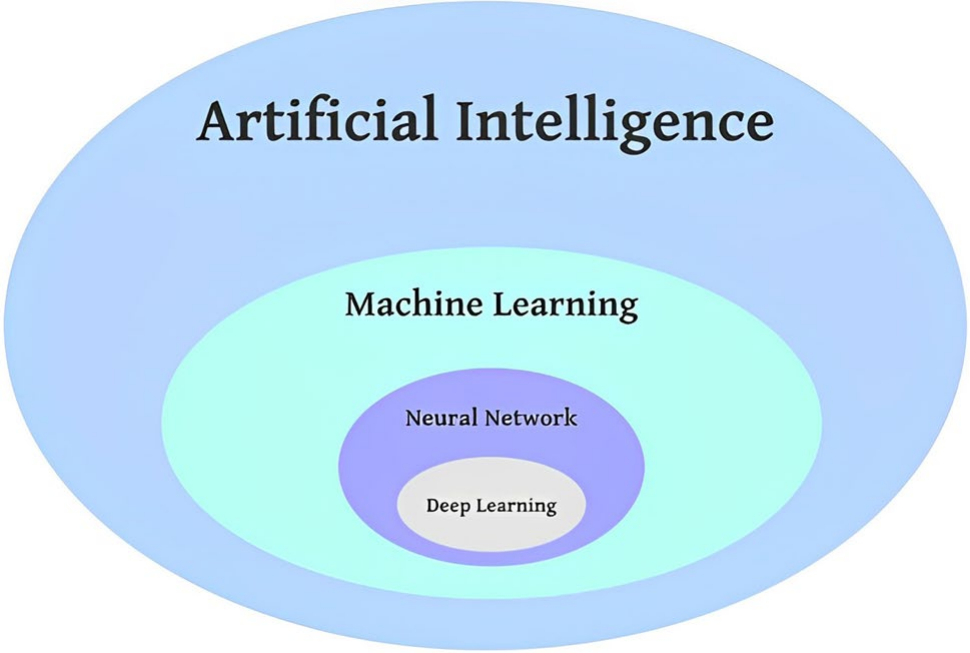
Relationship between AI, ML, NN, and DL approach

AI is a vast area merged into various fields of mathematics and science. Everything a machine can do automatically that is considered "intelligence" would be a subset of AI [49]. AI algorithms are taught on population representation information [50, 51]. One of the most important subfields of AI is ML, and the essential subfields of ML are Neural Networks and DL (Fig. 4).

The ML’s goal is that the machine can train itself based on input data set, experience, and receiving information from feedback [52]. The ML algorithm optimizes itself based on the information received from the feedback to be as accurate as possible in a particular task. Ideally, the ultimate goal is that it should work accurately on new unseen data sets as well [53].

Imaging source in the medical area is one of the most widely-used tools for diagnostic patient information. Still, it relies on human interpretation and is subject to increasing resource challenges. Automatic diagnosis of medical imaging through AI, especially in the field of DL, has effectively solved the problems of human error caused by inaccuracy or lack of sufficient experience. AI also plays a crucial role in image-based disease classifications, computer-aided diagnosis (CAD), and image disease segmentation. Since tissues and organ images in the healthcare system cannot be accurately simulated with simple equations, diagnosis tasks in medical imaging need to be learned through a training process.

Detection of any disease and prevention of its spread requires continuous checking and reviewing of data. Prompt action based on accurate data has a significant social and financial impact on the lives of people around the world [51]. The use of AI in healthcare has improved the collection and processing of valuable data and, at higher levels, the programming of surgical robots [54]. AI describes a machine's power to study how a human learns by image recognition and pattern recognition in a problematic situation. AI in health care has changed how information is collected, analyzed, and developed for patient care [55].

### Machine learning application in diagnosis image-based diseases

ML algorithms have many applications in various fields [56–58]. As a subfield of AI in medical imaging analysis, ML is a promising and growing field. ML has broad applications in computer vision, computer-aided diagnosis, and image processing in detecting diseases [59]. As medical imaging has advanced with the introduction of new imaging techniques such as multiple incision CT, positron emission tomography, tomosynthesis, magnetic resonance imaging, tomography, and diffuse optical tomography, progressive ML methods are increasingly needed for medical imaging analysis. ML consists of a set of plans for automatically detecting patterns in data and then using those methods to predict future data or make decisions in uncertain situations. The most distinctive feature of ML is that it is data-driven, with limited human participation in the decision-making process. The program learns by analyzing training data and making predictions when new data is entered [60].

Several recent techniques in ML have been applied to predict or diagnose diseases [61, 62]. Natural Language Processing (NLP) techniques have been used to analyze electronic health records (EHRs) to extract information that can be used to predict or diagnose diseases [63]. Explainable AI techniques, such as SHAP (SHapley Additive exPlanations), are presented to interpret the predictions made by ML models, which is essential in a medical context where the decision-making should be transparent [64]. Generative models such as GANs (Generative Adversarial Networks) are invented to generate synthetic medical images that can augment existing data, such as lung disease, and improve the result performance [65]. These techniques are not mutually exclusive and can be combined to improve the model's performance. The technique selection depends on the data type and the specific problem [66].

### Deep learning applications in diagnosis image-based diseases

DL is the most powerful technology that can automatically learn several features and patterns, making itself one of the most vigorous techniques. DL has made it possible to build predictive models for the early diagnosis of diseases. As scientists use proven pattern analysis methods, DL algorithms perform better than traditional ML methods because of the highly accurate results, automatic feature extraction, and massive data analysis. When it comes to big data, the results of using DL algorithms show a clear advantage over ML. Moreover, the predictive performance of DL often surpasses humans to recommend DL as the preferred method for dealing with images [67]. DL has received exceptional articulation in the medical field regarding image processing because the diagnosis primarily focuses on extracting useful information from images. In medical image-based diagnosis, DL algorithms are mainly of various types, including CNN, Deep Neural Network (DNN), Deep Belief Network (DBN), Deep Automatic Encoder, Deep Boltzmann Machine (DBM), Deep Intense Normal Machine Learning (DC-ELM), recursive neural network (RNN), and their types such as BLSTM, MDLATM [68]. Also, RAGCN (Region Aggregation Graph Convolutional Network) is a DL technique for analyzing medical data that utilizes graph convolutional networks (GCNs) to aggregate information from different regions of an image. It is specifically designed for medical images, such as CT and MRI scans, which often have multiple regions of interest (ROIs) that need to be analyzed separately. RAGCN uses a graph-based approach to segment the image into different regions and then applies GCNs to each part to extract features and make predictions. The authors in [69] presented an automatic bone age estimation method using CNN and GCN. They used CNN and GCN for feature extraction and inference of bone key regions, respectively. By combining these two types of networks, they were able to design a new GCN (RAGCN) that can investigate the features of the region in bone age assessment.

Lesion-attention pyramid network (LAPNet) is another DL medical data method designed to detect and classify lesions in medical images. LAPNet uses a pyramid-based architecture to extract features from the image at different scales. It also uses an attention mechanism to focus on specific regions of the image that are likely to contain lesions; authors in [70] used this technique to grade diabetic retinopathy. They trained LAPNet on a large dataset of medical images to learn to detect lesion regions.

These are some examples of the various DL techniques that are being used to predict or diagnose diseases. It is important to note that the field of DL is constantly evolving, and new techniques are being developed or combined all the time.

## Discussion

### The effective role of AI technologies in identifying and predicting human disorders and diseases

AI technologies are increasingly used in medicine to succeed and gain more accurate knowledge about dangerous disorders and diseases [71, 72]. Since AI can interact constructively with image data in the medical world, it is increasingly used in disease diagnosis and prediction [73].

Learning algorithms and big data derived from medical records or wearable devices are the two most vital tools to implement AI methods efficiently in the health care system to improve disease diagnosis, disease classification, decision-making processes activities, walking aids performance, providing optimal treatment choices, and ultimately helping people to live safer and more prolonged. AI is used to enhance medical analysis and diagnosis in a short time [74, 75]. For instance, this technology can detect dangerous tumors in medical images, allowing pathologists to diagnose the disease in the early stage and treat it instead of sending tissues or lesions samples to a lab for long-term investigation [76]. AI-based algorithms are an effective tool for identifying undiagnosed or less-diagnosed patients, unencoded, and rare diseases. Thus, AI models for disease diagnosis provide ample opportunity for early diagnosis of patients [76].

The application of ML and DL techniques to diagnose heart diseases is increasing significantly. Due to the existence of a wide range of medical imaging methods, such as CT, ECG, and echocardiography in cardiology, DL can be used accurately and advanced in the analysis and review of cardiovascular data [77–79]. Coronary atherosclerotic heart disease is a common cardiovascular disease that causes disabilities and severe morbidities. Early diagnosis of this disease is highly effective and has an impressive impact on treatment. In this term, ML and DL methods have achieved remarkable progress in coronary atherosclerotic heart disease diagnosis [80]. For instance, CT-Fractional Flow Reserve (CT-FFR) based on ML can simplify the processing of diagnosis and reduce times, which would be a powerful tool for predicting major adverse cardiac events [81, 82]. Also, CT-FFR based on the DL can simplify the computation, reduce time, and enhance prediction [83, 84]. Researchers have used SVM and ANN methods for the early diagnosis of various heart diseases in 170 patients [85]. They examined Arrhythmia, Cardiomyopathy, CHD, and CAD through SVM and ANN models. The SVM algorithm resulted in 89.1%, 80.2%, 83.1%, and 71.2% accuracy for Arrhythmia, Cardiomyopathy, CHD, and CAD, respectively. Likewise, the ANN algorithm resulted in 85.8%, 85.6%, 72.7%, and 69.6% accuracy for Arrhythmia, Cardiomyopathy, CHD, and CAD, respectively. Another study was proposed to predict coronary heart disease (CHD) and used the South African Heart Disease dataset of 462 samples [86]. To diagnose and improve the prediction rate of CHD, they used three supervised learning techniques, Naïve Bayes, SVM, and Decision Tree. In their study, the accuracy with library data was 83.9% for cardiovascular diseases, and for diabetes, the accuracy with library data was 95.7%. Another study on different ML techniques for predicting CHD showed that the SVM classifier achieved 95% accuracy and is superior to other ML methods [87]. Furthermore, other researchers investigated the predictive power of SVM, ANN, and Decision Tree algorithms on CHD disease for 502 samples [88]. The accuracy results of these three algorithms showed that SVM surpassed the other algorithms with an accuracy of 92.1%.

AI's massive applications in medical fields have also provided accurate prediction and detection of brain diseases. Recent ML and DL approaches are mainly used in diagnosing various brain and neurodegenerative diseases such as Alzheimer's disease (AD), Parkinson's disease (PD), and brain tumor, which has always been very difficult to detect in the early stage [89]. AI has made it possible to process and analyze a massive amount of brain signals and data to discover insights and correlations which are not completely obvious to the human eye. The most widely used algorithm for disease detection is DL-based CNN models [90, 91]. A recent study examined pre-trained models for predicting and detecting Alzheimer’s disease [92]. In this study, the EfficientNetB0 model outperformed other models and obtained an accuracy of 92.98%. A combination of different AI algorithms was used for the early diagnosis of Parkinson’s disease recently [93]. The best result was obtained by combining the genetic algorithm and random forest with an accuracy of 95.58%, which in turn is the best result in this field in recent works.

AI’s advanced algorithms also greatly assist in detecting and predicting breast cancer in the early stages. Breast cancer is a deadly disease among females that causes the deaths of millions of people annually [94]. However, diagnosis in the early stages has a vital role in treating and controlling. The Wisconsin Breast Cancer Dataset (WBCD) is a widely-used dataset for researchers investigating ML methods to diagnose breast cancer. The least-squares support vector machine (LSSVM) algorithm was successfully applied to WBCD to diagnose breast cancer and achieved 98.53% classification accuracy [95]. Also, a hybrid fuzzy-artificial immune system with a k-nearest neighbor algorithm was proposed on the WBCD and resulted in 99.14% classification accuracy [96]. An SVM algorithm combined with feature selection was used to diagnose breast cancer using WBCD and obtained 99.51% classification accuracy [97]. In another work, an optimized SVM was proposed to diagnose breast cancer prognosis based on the WBCD dataset [98]. This method was evaluated in two stages and obtained 96.91% and 97% accuracy, respectively. Another study presented a combination of three classifiers of SVM, K-nearest neighbors, and probabilistic neural networks to detect benign and malignant breast tumors [99]. They achieved 98.8% and 96.33% accuracy against two benchmark datasets.

Neural fuzzy methods, K-nearest neighbor, quadratic classifier, and their combination have been used to classify and diagnose breast cancer [100]. The resulting accuracy in fuzzy neural methods, KNN, quadratic classifier methods, and their combination method are 94.28%, 96.42%, 94.50%, and 97.14%, respectively. In this work, the association method provides more accurate results than every single model. A mammography-based machine learning classifier (MLC) was performed for breast cancer diagnosis to classify segmented regions on craniocaudal (CC) and/or mediolateral oblique (MLO) mammography image views [101]. They gained the area under the ROC 0.996 curve when combining features from CC and MLO views. Also, in another work, the C4.5 algorithm was used to classify the breast cancer dataset from SEER into two groups carcinomas in situ and malignant potential [102]. In the training phase, an accuracy of ~ 94% and ~ 93% in the testing phase was obtained. In a comprehensive study, several classifiers, including different classifiers decision tree (J48), Multi-Layer Perception (MLP), Naive Bayes (NB), Sequential Minimal Optimization (SMO), and Instance-Based for K-Nearest neighbor (IBK) were used to diagnose and predict breast cancer in three databases: Wisconsin Breast Cancer (WBC), Wisconsin Diagnosis Breast Cancer (WDBC) and Wisconsin Prognosis Breast Cancer (WPBC) [103]. This study used the classification accuracy and confusion matrix based on tenfold cross-validation. The integration of SMO, J48, NB, and IBK was associated with 97.2818%, 97.7153%, and 77.3196% accuracy for the WBC, WDBC, and WPBC datasets, respectively. Researchers proposed multiple data mining methods in another study to diagnose and predict breast cancer using the UCI machine learning and SEER datasets [104]. The results showed that the decision tree as the best predictor achieved 93.62% accuracy in both datasets.

ML methods, including neural networks, random forests, and support vector machines, have been used to predict and categorize genetic disorders from different amounts of genetic data. Scientists have faced challenges finding biomarkers for complicated genetic diseases due to their diverse genotypes. AI, especially ML and DL methods, could enhance the accuracy of predicting genetic disorders. For instance, the ANN-based model achieved 85.7%, 84.9%, and 84.3% for the training, testing, and validation phases, respectively [105]. The ML performance accuracy in psychiatry from genotypes varied between 48 and 95% [106]. Though genetic diseases need automated predictors based on AI, there are still some limitations in data sample size and a lack of high-standard models [105]. One of the challenges in genetic microarray analysis is identifying genes or groups of genes that are highly expressed in tumor cells but not in normal cells [107]. AI-based methods have provided a significant role in classifying cancerous microarray data. Three supervised ML techniques were proposed to organize gene data, including the C4.5 decision tree, bagged, and boosted decision trees. In this work, ensemble ML (bagged and grown decision trees) outperformed single decision trees [107]. Researchers also studied autism spectrum disorder (ASD), which has a genetic nature, using datasets of toddlers, children, adolescents, and adults to evaluate and determine the best-performing classifier of ASD. They found that MLP performed better than other classification algorithms and achieved 100% accuracy [108].

AI techniques also can have broad applications in dermatology. ML and DL can impressively be taught based on data to diagnose, predict, and classify the characteristics of various skin disease samples. However, dermatology science is still behind in accepting and using these advanced techniques. Detection in the early stages is an essential factor for effective skin cancer treatment. In this term, CNN-based algorithms can examine skin dataset images to diagnose skin cancer. Young specialists cannot always accurately detect skin cancer due to a lack of experience or human errors. So, developing automated systems based on AI can help them significantly diagnose skin diseases to save patients' lives and reduce financial costs [109]. Researchers used two ML-based strategies, ensemble learning, and DL, to analyze skin cancer lesions [110]. In this work, the DL approach outperformed the ensemble learning, for prediction demonstrated an accuracy of 91.85% and for classification of skin cancer resulted in 90.1% accuracy. The combination of Bayesian DL and an active learning approach has been used to diagnose skin cancer [111]. This approach achieved the best performance in ISIC 2016 with 75% accuracy.

The interaction of digital pathology and AI has led researchers to examine datasets more accurately and provide precise results for prostate cancer diagnoses. A vital treatment for prostate cancer is radiotherapy, but its toxicity recognition is problematic for various individuals [112]. In this case, AI could provide proper insights on predicting how a patient will react to the different therapy methods. Furthermore, AI-based technologies have demonstrated acceptable accuracy in diagnosing prostate lesions and predicting prostate cancer, patient survival rate, and treatment response. Researchers developed a supervised AI-based model for the diagnosis of prostate cancer in the early stage [113]. They used MRI images labeled with histopathology information which resulted in 89% accuracy in classification. Another study proposed a novel DL approach named XmasNet to classify prostate cancer lesions using 3D multiparametric MRI data provided by the PROSTATEx in the training phase [114]. XmasNet outperformed ML classical methods with an AUC of 0.84.

Different techniques have been proposed to detect lung cancer in the early stages; most are based on CT scan images, some utilizing x-ray images. Although it is challenging to catch it in the initial stage, it has been proven that early detection improves the survival rate of lung cancer patients [115]. Computer-assisted diagnosis (CAD) based on a DL-based framework for lung cancer diagnosis was proposed for the Kaggle Data Science Bowl 2017 challenge and placed 41 out of 1972 teams with high accuracy [116]. Deep CNN (DCNN) was used to classify different types of lung cancer into adenocarcinoma, squamous cell carcinoma, and small cell carcinoma [117]. They examined the probabilities of these three types of cancers using three-fold cross-validation and obtained 71% accuracy. This model consisted of three convolutional layers, three pooling layers, and two fully connected layers. Another research compared three deep neural networks model (CNN, DNN, and SAE) to diagnose and classify benign and malignant lung nodules using the LIDC-IDRI database [118]. CNN obtained the best performance among these three networks, with an accuracy of 84%. A comprehensive study examined three DL-based algorithms, including convolutional neural network (CNN), deep belief network (DBN), and stacked denoising autoencoder (SDAE), to diagnose lung nodules in CT images [119]. The best performance of the area under the curve (AUC) was 0.899 ± 0.018 achieved by CNN.

Diagnosis and prescribing medicines in the early stage are essential keys to treating respiratory infections. AI algorithms can assist healthcare experts in detecting and analyzing pulmonary diseases. A DL-based CNN model was presented to analyze the respiratory audio data for Chronic Obstructive Pulmonary detection and achieved 93% accuracy [120]. Also, a framework model based on CNN was proposed to diagnose Covid-19 disease using X-ray images. In this work, they achieved an accuracy of 95.7% [121].

## Challenges, potential solutions, and future prospects of AI methods

AI has a broad role in healthcare systems for diagnosis, prediction, and prevention purposes. However, several challenges exist in using DL and ML techniques in disease diagnosis and prediction. One of the significant challenges in AI algorithms is the need for massive data in training phases which is not always practical in most diseases. Another challenge is labeling data, which requires professionalism and expertise, and is time-consuming and highly costly [122]. This can make it challenging to develop accurate models for rare or new diseases. One potential solution to the lack of labeled data is to use techniques such as data augmentation, which can be used to increase the size of the dataset artificially [123].

The complexity of computation and architecture in DL-based model is another challenge in this area. One potential solution for reducing the complexity of computation and architecture in DL models is to use model compression techniques such as pruning, quantization, and low-rank factorization [124]. These techniques can help reduce the number of parameters and computational resources required while maintaining good performance. Analysis of low-contrast images is also a challenging mission to examine patterns and features. One of the enhancement techniques used for boosting the contrast is Histogram Equalization (HE). An ML-supervised method based on hyperparameter selection using the HE technique was proposed to improve the visual appearance and increase image contrast while keeping its natural aspect [125]. Other researchers also proposed a new approach for contrast optimization based on HE in cancer diagnosis using ultrasound medical imaging [126].

Even though various researchers have recently addressed the issue of model complexity in DL [127–130], there is still a need for further investigation and effort in this area [131]. Through DL or ML algorithms, a constraint on the dataset is likely to create a similarly constrained model. For example, outside the pre-defined boundaries, the network may appear useless because it has not been taught how to handle such instances.

Also, more efforts are needed from medical community to convince future perspectives and acceptance of AI technologies in diagnosing and predicting various diseases. Additionally, patient privacy must be taken seriously when entering data into artificial intelligence systems [132]. Therefore, global coordination and monitoring should be done, leading to the widespread and verifiable use of AI in healthcare procedures.

In particular, ML and DL can be used to analyze large amounts of medical data, such as patient records, imaging studies, and laboratory results, in order to identify patterns that might not be obvious to human doctors [133]. This can lead to more accurate and efficient diagnosis and the ability to predict which patients are at the highest risk for certain conditions. Additionally, these techniques can be used to develop personalized treatment plans for individual patients based on their unique characteristics and medical history [134, 135]. Also, it is vital to ensure the data used to train these models is diverse and unbiased to avoid any inaccuracies or discrimination in diagnosis and predictions [136].

In the future, ML and DL algorithms will continue to improve and become more widely adopted in the healthcare industry, leading to better disease prediction and diagnosis for patients. ML and DL techniques can be used to analyze genomic data to identify genetic markers associated with different diseases, which could lead to more precise diagnosis and personalized treatment plans. Another promising area for these techniques in healthcare is in the development of predictive models for disease progression and treatment response. These models could help physicians to identify patients at high risk for complications, or those who are unlikely to respond to certain treatments, allowing for early intervention and more effective care. As these techniques continue to advance, we can expect to see even greater improvements in patient care in the future.

## Conclusion

DL and ML techniques have strong potential to revolutionize the field of disease diagnosis and prediction. In diagnosing the disease, the accuracy and correctness of the diagnosis is the most critical factor in the treatment process. AI has proven significant accuracy in the detection of image-based diseases as well as in the prediction of treatment outcomes regarding survival rate and treatment response. The enormous quantity of image data requires implementation into processing phases through immediate, reliable, and accurate computing power provided by AI methods. In diagnosing diseases, issues such as accuracy in detection, effective treatment, and ensuring the well-being of patients are critical. AI includes vast and diverse data, algorithms, deep computing methods, various neural networks, and emerging techniques constantly evolving to meet human needs. This study aims to investigate the performance of AI techniques in diagnosing and predicting various diseases. According to the findings of this research, SVM has the best performance for predicting heart diseases. Supervised DL networks, such as CNN-based models, are widely used due to their high accuracy and fast image recognition, especially for diagnosing in respiratory, lung, skin, and brain diseases which have led to significant results. For breast cancer diagnosis, usually combining KNN with other networks, such as SVM, leads to high accuracy in diagnosis. Therefore, DL and ML, with impressive experimental results in detecting and classifying medical images, significantly impact the success of many diseases discussed in this study. In other words, AI-based methods assist medical systems in diagnosing and predicting conditions by optimizing the use of different resources. Also, with the rapid development of AI technologies, the objective diagnosis of various diseases will no longer be an uphill task for doctors in the near future.

## Data Availability

Data sharing does not apply to this article as no datasets were generated or analyzed during the current study. All authors, Nafiseh Ghaffar Nia, Erkan Kaplanoglu, and Ahad Nasab have approved all the statements in this work.
